# Probing the Highly Disparate Dual Inhibitory Mechanisms of Novel Quinazoline Derivatives against *Mycobacterium tuberculosis* Protein Kinases A and B

**DOI:** 10.3390/molecules25184247

**Published:** 2020-09-16

**Authors:** Fisayo A. Olotu, Mahmoud E. Soliman

**Affiliations:** Molecular Bio-Computation and Drug Design Laboratory, School of Health Sciences, University of KwaZulu-Natal, Westville Campus, Durban 4001, South Africa; olotuf@ukzn.ac.za

**Keywords:** *Mycobacterium tuberculosis*, multi-drug and extensively drug resistance, dual-targeting, serine/threonine protein kinases, structure-based design, medicinal chemistry

## Abstract

*Mycobacterium tuberculosis* (*Mtb*) serine/threonine (Ser/Thr) Protein kinases A (PknA) and B (PknB) have been identified as highly attractive targets for overcoming drug resistant tuberculosis. A recent lead series optimization study yielded compound **33** which exhibited potencies ~1000 times higher than compound **57**. This huge discrepancy left us curious to investigate the mechanistic ‘dual’ (in)activities of the compound using computational methods, as carried out in this study. Findings revealed that **33** stabilized the PknA and B conformations and reduced their structural activities relative to **57**. Optimal stability of **33** in the hydrophobic pockets further induced systemic alterations at the P-loops, catalytic loops, helix Cs and DFG motifs of PknA and B. Comparatively, **57** was more surface-bound with highly unstable motions. Furthermore, **33** demonstrated similar binding patterns in PknA and B, involving conserved residues of their binding pockets. Both π and hydrogen interactions played crucial roles in the binding of **33**, which altogether culminated in high Δ*G_s_* for both proteins. On the contrary, the binding of **57** was characterized by unfavorable interactions with possible repulsive effects on its optimal dual binding to both proteins, as evidenced by the relatively lowered Δ*G_s_*. These findings would significantly contribute to the rational structure-based design of novel and highly selective dual inhibitors of *Mtb* PknA and B.

## 1. Introduction

The increase in the emergence of multi-drug and extensively drug resistant (MDR/XDR) *Mycobacterium tuberculosis* (*Mtb*) strains represents a major setback to the treatment of tuberculosis, which is thereby still as a major global threat [1]. As reported, about two million new TB-related cases and deaths occur each year, in addition to a very worrisome approximately two billion latent infections [2]. Since the efficacies of existing anti-tubercular drugs have been thwarted by increasing incidences of drug resistance, there is the need for novel drugs that could disrupt crucial pro-resistance *Mtb* machinery.

In recent years, *Mtb* serine/threonine protein kinases (STPKs) have been pinpointed for their concerted involvement in mycobacterial viability [3,4]. This family of mycobacterial protein (11-membered) mediates protein phosphorylation events which play key roles in signal transduction to downstream pathways; typical of the two-component signaling cascade [5,6]. This underlies intracellular regulatory mechanisms by which the bacterium responds to extracellular signals and adapt to changes in environmental conditions. Usually, post-translational modifications such as phosphorylation, ubiquitination, and acetylation describe mechanisms by which *Mtb* responds to oxidative stress induced by external factors [7,8].

Similar to eukaryotic kinases, STPKs possess extra cytoplasmic regions; to which extracellular molecules bind and intracellular kinase domains that phosphorylate a cohort of substrate proteins resulting in the activation of signaling cascades and eventual alterations in the expression of cellular biomolecules such as genes, proteins, lipids among others [5,6,9]. Members of the *Mtb* STPK family include protein kinases A–H, J–L; PknA, PknB, PknC, PknD, PknE, PknF, PknG, PknH, PknJ, PknK and PknL, which drive various microbial processes such cell wall synthesis, cellular growth, division, development, metabolism, and dormancy [5,10,11,12,13]. Most prominent among the STPK protein family are PknA and PknB, which have been implicated in the regulation of cell wall synthesis, resuscitation from dormancy, transcription, translation, and other processes that strongly enhance adaptation to environmental stress, bacterial cell growth and division [9,14,15,16,17,18,19,20].

Protein kinases A and B are encoded by *pknA* and *pknB* genes, respectively, which are also located on the same operon containing genes that code for a Ser/Thr phosphatase (*pstP*), class B penicillin-binding protein (*pbpA*) and candidate peptidoglycan (PG) synthase (*rodA*) [21,22]. PknA is made up an intracellular kinase domain that is connected to an extracellular domain via a juxtamembrane domain, likewise PknB which consists of an intracellular N-terminal and an extracellular C-terminal domain composed of four PASTA domains [23,24]. Crucial to the activity of these proteins are various structural elements that constitute their nucleotide binding regions such as the P-loop, catalytic loop, activation loop, helix C and the DFG motif. Helix C is located in the linker segment that connects the kinase homology domain to the transmembrane helix. The P-loop is a hexapeptide (PknA: 20-ATGGMG-25 and PknB: 18-GFGGMS-23) that coordinate phosphates via the main chain glycine amides [23]. The catalytic domain consists of the ATP binding site while the activation loop functions in the coordination of the Mg2+ or Mn2+ ion through specific inter-residual contacts [23,25]. Also contained in the activation loop are phosphorylated residues (PknA: A169, T172, T174, T176 and PknB: S166, S169, T171 and T173), although highly disordered [26]. Also, several structural features have been associated with the catalytic domain of these proteins with respect to ligand or substrate binding such as the tilting away of the helix C from the active site or the DFG out or in conformations [27,28,29,30]. 

The significance of *Mtb* PknA has been attributed to its role in the mechanics and regulation of *Mtb* cellular shape as supported by its upregulation during the exponential growth and infection phases of the mycobacteria [31]. Moreover, its autonomous activation enhances in vitro growth and survival relative to its phosphorylative effects on downstream proteins involved in cell division, peptidoglycan, and mycolic acid synthesis [32,33].

The crucial role attributed to *Mtb* PknB centers on its ability to enhance and sustain mycobacterial growth [4,34]. Also, its essential involvement in the reactivation of mycobacterial cells from the hypoxic state has been previously reported [13]. More so, Ser/Thr phosphorylation of proteins such as GarA regulatory protein, MabA, KasB, and InhA informs the ability of PknB to regulate *Mtb* central carbon metabolism and mycolic acid synthesis [15,35,36,37,38].

Moreover, PknA and PknB have been proposed as attractive therapeutic targets for inhibiting both active and latent forms of tuberculosis [13,26]. This is due to their critical involvement in sustaining bacterial growth as experimented in culture medium and host macrophages infected with *Mtb* [10,12,14,23,39,40]. Interestingly, these proteins have less than 30% similarity with eukaryotic kinases in primary amino acid sequences presenting an avenue for achieving selective therapeutic targeting over human host kinases [23,28].

**Figure 1:**
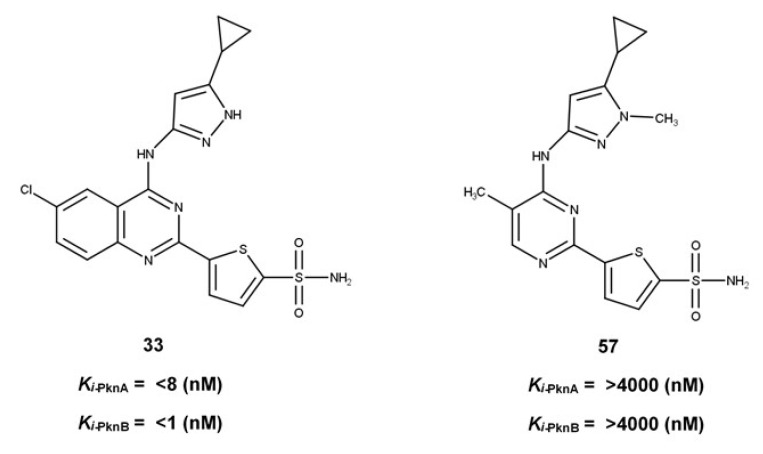
Chemical structures of *Mtb* Ser/Thr Protein kinases A and B inhibitors with their experimentally reported *K_i_* values.

Although numerous research efforts have been directed towards inhibiting either of these proteins, a recent paradigm shift has been aimed at the development of dual selective inhibitors that can simultaneously target both proteins [4,8,26,34,41,42]. This therapeutic approach presents an avenue to minimize the rate at which resistance occurs while at the same time increasing specificity. In a recent study, Tiansheng and co-workers [26] synthesized a series of quinazoline derivatives with varying degrees of inhibitory potencies against protein kinases A and B. Significant among these series was the dually selective compound 5-(6-chloro-4-((5-cyclopropyl-1*H*-pyrazol-3-yl)amino)quinazolin-2-yl)thiophene-2-sulfonamide (**33**) with Ki values <8 nM and <1 nM for PknA and PknB, respectively [26]. This compound exhibited the highest inhibitory activity and selectivity compared to other derivatives (Figure 1). Interestingly, a chemical analog, (4-(5-cyclopropyl-1-methyl-1*H*-pyrazol-3-ylamino)-5-methylpyrimidin-2yl)thiophene-2-sulfonamide (**57**) [5], exhibited poor selectivity and inhibitory potency against these kinases (Ki > 4000 nM). It is therefore expedient to understand the mechanisms by which chemical substitutions on these derivatives mediate significantly disparate selective and inhibitory activities on *Mtb* PknA and PknB. 

Herein, we implemented qualitative GPU-accelerated molecular dynamics (MD) simulations, binding free-energy calculations and free energy decomposition analysis to: (i) probe the dynamics of selectivity and non-selectivity elicited by **33** and **57**, respectively, towards PknA and PknB; (ii) investigate the ligand-induced dynamics of the alternative hinge binding region and (iii) study how chemical substitutions influence the binding affinities of both compounds. To achieve this, we prepared and studied six protein systems: unbound PknA, unbound PknB, **33**-bound PknA, **33**-bound PknB, **57**-bound PknA and **57**-bound PknB. The respective compounds were bound to the nucleotide binding pockets of the proteins which were defined in line in previous crystallographic studies [26,42]. This region consists of various structural elements as earlier mentioned which exhibits various conformational variations with respect to the binding of substrates or small molecular compounds.

We expect that rational insights from this study would enhance further lead optimization that could open up avenues for the discovery of novel compounds with improved selectivity and dual inhibitory potencies against PknA and B in anti-TB therapies.

## 2. Results and Discussion

### 2.1. Conformational Stability and Ligand-Induced Variations 

Structural arrangements of a protein play a critical role in its biological functionalities. Also, the ability of small-molecule compounds to mediate dual selective inhibition of protein homologs could be enhanced or limited by the nature of the respective target sites. Moreover, structural insights into binding site architectures could provide a rational explanation for the mechanistic activities of small therapeutic molecules which could also enhance the structure-based design of novel chemical entities.

Structural and sequential disparities among bacterial kinases of the STPK family have been widely described, which underlies our focus on the conformational dynamics of PknA and PknB, particularly the catalytic domains (nucleotide binding pockets), relative to the binding of **33** and **57**. This presents an approach to understand how the selective inhibitory activities of the compounds varied from PknA to PknB as reported by Wang et al. [26].

Firstly, we employed the RMSD metrics to enumerate the stability of the studied systems, which could be deduced from motions of the constituent Cα atoms across the simulated trajectories. 

**Figure 2:**
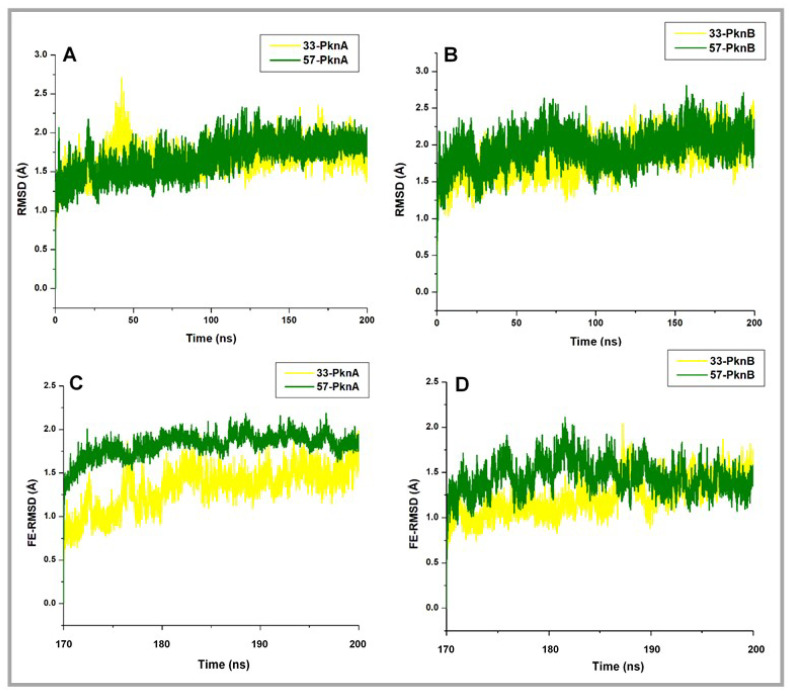
RMSD plots showing variations across the entire PknA and PknB structures as induced by the binding of **33** and **57**. Finally equilibrated RMSD (FE-RMSD) plots estimating minimal Cα deviations for the ultimate 30 ns time frames are also shown. (**A**) Whole structure RMSD plot for **33**- and **57**-bound PknA (**B**) Whole structure RMSD plot for **33**- and **57**-bound PknB (**C**) FE-RMSD plots for **33**- and **57**-bound PknA (**D**) FE-RMSD plots for **33**- and **57**-bound PknB.

From the resulting plots (Figure 2A,B), we observed that the systems seemingly converged with minimal deviations at about 170ns with conformational RMSDs < 2 Å. Then, the finally equilibrated RMSD (FE-RMSD) of the proteins’ backbone Cα atoms were defined from the terminal 30ns where minimal Cα motions were observed across the systems. These post-equilibrated time frames were also employed for subsequent analyses. 

Results are presented in Figure 2C,D, and from our findings, we predict that structural fluctuations may follow the **57**-PknA > **57**-PknB > **33**-PknA > **33**-PknB order, a presumption that would be subsequently investigated using other methods (metrics). Interestingly, estimated mean values, as reported in Table 1 may correlate with experimental bioactivity data and showed that the lowest FE-RMSD value was obtained for **33**-bound PknB which reportedly had the better *Ki* value. Further FE-RMSD estimations revealed that the unbound proteins (PknA and PknB) demonstrated high structural deviations relative to their ligand-bound forms (Supplementary Figure S1).

Also, hydrogen bond (HB) analyses were performed to further determine the stability of the studied systems with respect to ligand binding (Supplementary Figure S2). Considering the overall systems, findings revealed that average HB increased in the presence of the compounds, with a higher proportion in the **33**-bound proteins. Estimated HBs were 149, 143, 132 and 126 respectively for **33**-PknB, **33**-PknA, **57**-PknA, **57**-PknB complexes. Higher HB counts associated with the **33**-bound complexes could deduce an increase in the stability of the proteins [43,44]. Relatively, reduced average HBs (PknA = 122, PknB = 123) in the unbound proteins could possibly indicate an increase in structural flexibility or instability [43,45].

In addition, HB interactions with occupancies ≥90% were selected from the ultimate 30 ns trajectories (170–200 ns) and compared among the systems to gain further clues into the relative stabilities of the proteins (Supplementary Table S1). As presented, 7 HBs had occupancies >90% in unbound PknA, and 5 in unbound PknB, which increased to 14 (**33**-PknA), 15 (**33**-PknB) and 10 (**57**-PknA and B). These additional HBs may account for the stability of the proteins at these ultimate time frames. Taken together, we can suggest that the binding of compounds **33** and **57** stabilized the overall proteins’ structure relative to their unbound forms.

**Figure 3:**
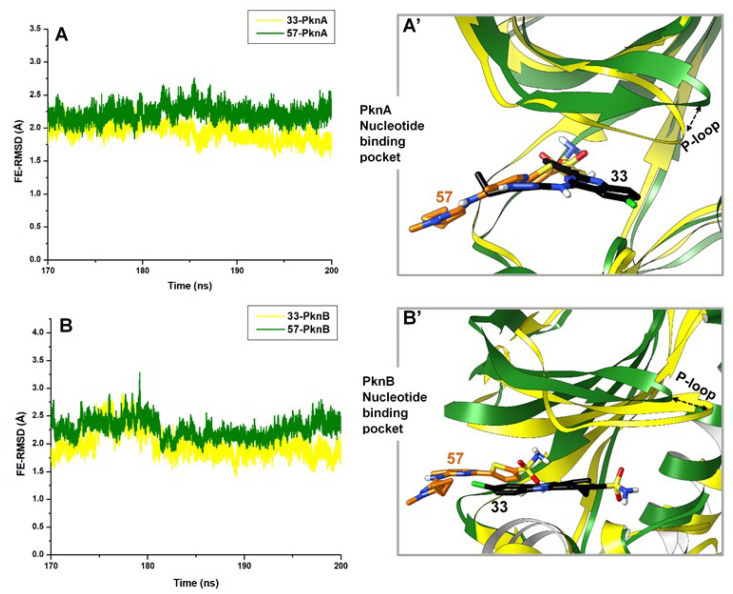
FE-RMSD plot showing systematic Cα deviations as induced by **33** and **57** at the nucleotide binding pockets of (**A**). PknA and (**B**). PknB. Orientations of the compounds with respect to their binding activities are shown in (**A’**,**B’**). Also captured are the alterations at the NBP P-loop induced by the binding of compound **33**.

Furthermore, using the post-equilibrated time frames (final 30 ns), we enumerated the degree of atomistic deviations induced differentially by the compounds at the NBPs of the proteins. Our findings revealed that the binding of **57** induced alterations in the structural arrangements of both PknA and B NBPs while the pockets were more stable in the presence of **33** (Figure 3). Estimated average RMSD values are also presented in Table 1. Hence, we could suggest that the dual binding of **33** to both proteins reduced residual mobility the active site regions as well, compared to compound **57**. These structural effects, as caused by **33**, could be as a result of high-affinity interactions with certain active site components, as elucidated in subsequent sections. In addition, the NBPs of unbound PknA and PknB demonstrated higher deviations which could suggest high mobility among constituent residues (structural components) relative to their catalytic activities.

**Figure 4:**
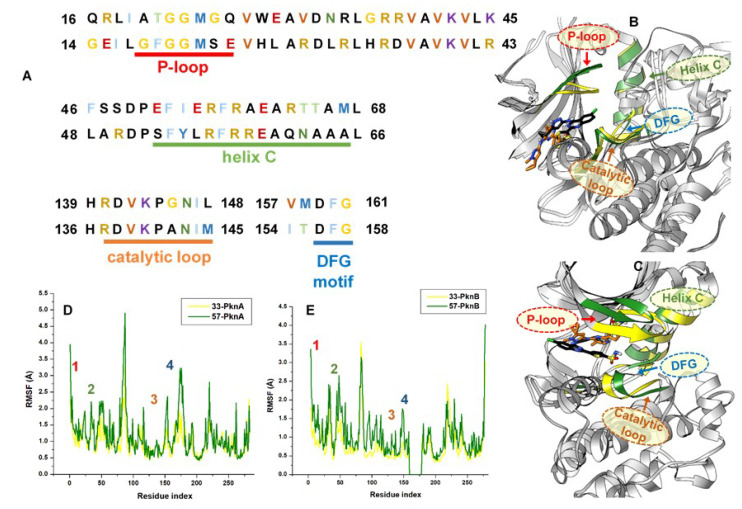
RMSF plots showing per-residual fluctuations across key components of PknA and PknB structures. (**A**) Sequence alignment of the NBP region of both proteins showing variations in constituent residues. Highlighted are the various residue sequences that encompass the P-loop (red), helix C (green), catalytic loop (brown) and DFG motif (blue). These regions are color-indicated on the RMSF plot in (**D**,**E**) (1 = P-loop, 2 = helix C, 3 = catalytic loop, 4 = DFG motif). 3D structural alignment of **33**- (black) and **57**- (orange) bound PknA and PknB are shown respectively in (**B**,**C**).

To gain further insights into the systemic alterations induced by the compounds, we employed the Cα RMSF metrics which is able to determine the extent of fluctuation of individual amino acid residues over the MD simulation period. We estimated the final equilibrated RMSF (FE-RMSF) depictive of the post-equilibrated trajectories. FE-RMSF analysis revealed higher structural fluctuations for **57**-bound PknA and PknB compared to minimal fluctuations exhibited both proteins when bound by **33** (Figure 4D,E). From the estimations, FE-RMSF was decreased in the **33**-bound proteins compared to the relatively higher values obtained for those bound by compound **57** (Table 1). This corroborates the FE-RMSD data which further implies that the ligand activities with respect to structural fluctuation were in the **57**-PknA > **57**-PknB > **33**-PknA > **33**-PknB order. This means that the least fluctuation occurred in the **33**-bound PknB while structural fluctuation was higher in the 57-bound proteins. This could be correlated with the experimental data which revealed that **33** was most active in PknB. Also, residual fluctuations were relatively higher in the unbound proteins compared to the ligand-bound systems. 

We further mapped out specific structural elements of both proteins; P-loop, helix C, DFG motifs and catalytic loops, since they are important to substrate binding and protein functionality [25,46,47]. The results, as presented in Table 2, revealed that the binding of **33** to PknA decreased motions at the helix C, catalytic loop and DFG motif. Also, motions were relatively reduced at the P-loop, catalytic loop and DFG motif by **33** in PknB. According to 3D-visualization of the structurally superposed **33**- and **57**-bound proteins, we observed that the P-loop, catalytic loop and, DFG-motif regions were extended into the active pocket in the presence of **33** with slight alterations in the helix C regions (Figure 4B,C). 

These variations in motions could be as a result of complementary ligand interactions with constituent residues as earlier mentioned. These binding dynamics would be discussed in subsequent sections. Comparatively, differences in the motions of these structural components relative to their unbound forms could be predictive of the degree to which they were altered with respect to ligand binding (Table 2 and Supplementary Figure S3). Accordingly, we could deduce that ligand binding induced a measure of structural rigidity among these components compared to the unbound forms. Also, between the **33**- and **57**-bound systems, we observed that less fluctuation were observed in the Helix C, catalytic loop and the DFG-motif which could indicate the ability of compound **33** to disrupt the catalytic activity of these kinases more potently.

Another important observation from Figure 3 was the surface-binding exhibited by compound **57** while **33** was bound deeply within the NBP of both proteins. These binding attributes exhibited by **57** could reduce its binding affinity since it is unable to access the deep pocket region of both proteins. 

**Figure 5:**
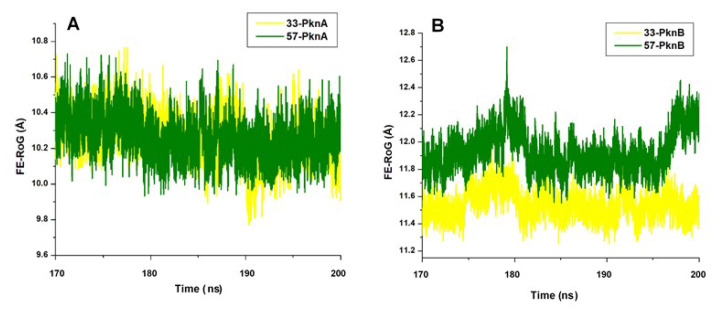
Cα RoG plot showing the degree of structural compactness at the NBP of (**A**) PknA-bound **33** and **57** (**B**) PknB-bound **33** and **57**.

We further estimated differential compactness of PknA and PknB NBPs (nucleotide binding pockets) in the presence of both compounds using the equilibrated radius of gyration (FE-RoG) metrics. This was employed herein to measure how the binding of compounds **33** and **57** alter the compactness of PknA and B active pockets and also corroborate variations among the various catalytic components as earlier estimated. RoG has been widely employed as a parameter to determine the compactness of protein systems which in turn could be used to suggest the structural stability (or mobility) of such systems [48]. It measures atomic distribution from the center of mass, hence a high RoG could correlate with high structural instability or motion and vice versa [49]. From the estimations, high mobility characterizes the unbound NBPs of PknA and B, as supported by their relatively high FE-RoG values (Supplementary Figure S1). However, the active pockets of PknA and B appeared to be more compact (less motions) in the presence of **33** relative to **57**. Prominent reductions in variations, as induced by **33**, could be due to alterations among constituent structural elements such as the P-loop, catalytic loop, and DFG-motif as shown in Figure 5. FE-RoG between **33**- and **57**-bound PknA-NBP varied by 0.99 Å while a difference of 3.39 Å was pertinent to **33**- and **57**-bound PknB-NBP (Table 1). These estimations could further reflect the dual bioactivity of **33** against PknA and PknB as experimentally reported.

### 2.2. Dynamic Cross Correlation Matrix (DCCM)

**Figure 6:**
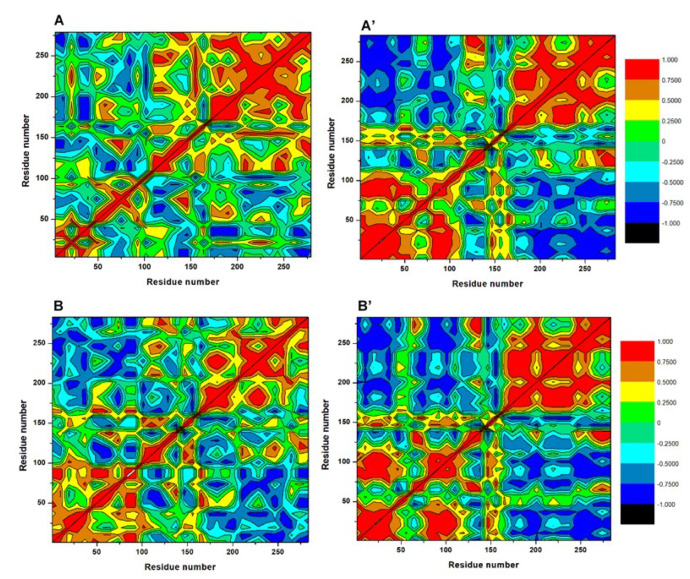
Dynamic cross-correlation matrices of the Ca fluctuations in the ultimate 30 ns simulation period (**A**) **33**-bound PknA, (**A’**) **57**-bound PknA (**B**) **33**-bound PknB and (**B’**) **57**-bound PknB.

The disparate Cα motions occurring among constituent residues of PknA and PknB when bound differentially by **33** and **57** were further described using the DCCM parameter. This analysis could also provide dynamical insights into the directions of residual motions along the MD simulation time frames. Resulting matrices are presented in Figure 6. Yellow to deep red colors depict positively correlated motions and cyan to black colors represent motions that are negatively correlated or anticorrelated. As shown, highly correlated motions occurred in unbound PknA while moderate → deep → negatively correlated motions (Supplementary Figure S2) were observed in unbound PknB, indicating high fluctuations and possibly, inter-residual interactions among constituent residues. Narrowing down, key structural components of their catalytic sites such as the P-loop, helix C and catalytic loop showed high correlations (PknA and B) which was reduced in the DFG motif of PknB (residues 156–158). Overall, highly correlated motions demonstrated by the unbound proteins could suggest their high elasticity or flexibility, a conformation that may be essential for their catalytic activities.

On the contrary, anti-correlated motions were increased in the presence of the compounds which could imply that their binding interfered with residual fluctuations, particularly at regions that constitute the NBPs of the proteins. Suggestively, this reduction in mobility could indicate restraints imposed by ligand binding. Comparatively, more correlated motions characterized the **57**-bound proteins as compared to the **33**-complexes which had more anticorrelated residual motions. Structural components of the NBPs were mapped out to further indicate the degree to which they were differentially altered by the ligands (Supplementary Figure S3C,D). As seen, anticorrelations were more enhanced in PknA around the P-loop, helix C, catalytic loop and DFG motif when bound by compound **33** while the DFG motif was more correlated in **33**-PknB. 

Moreover, since positive motions could indicate outward residual motions and vice versa [50], we could further indicate that the **33**-bound proteins were more stable in line with earlier RMSD and RMSF results. Likewise, we could suggest that the reduction in structural flexibilities of PknA and B, particularly the NBP, was induced by the ligands, since they appeared to be highly flexible in the unbound forms.

### 2.3. Systematic Analyses of Binding Dynamics to Understand Differential Inhibitory Mechanisms

We further probed the mechanisms by which both compounds mediated dual (in)activities against PknA and PknB over the entire MD simulation period. 3D conformations of the **33**- and **57**-bound complexes were retrieved from the resulting trajectories and subjected to comparative time-based analysis. The starting structures were the pre-MD **33**- and **57**-bound complexes and as observed the structural components were aligned with no alterations occurring yet. Based on our findings, a disparate fluctuation was observed, majorly at the P-loop region of PknA when differentially bound by **33** and **57**. 

**Figure 7:**
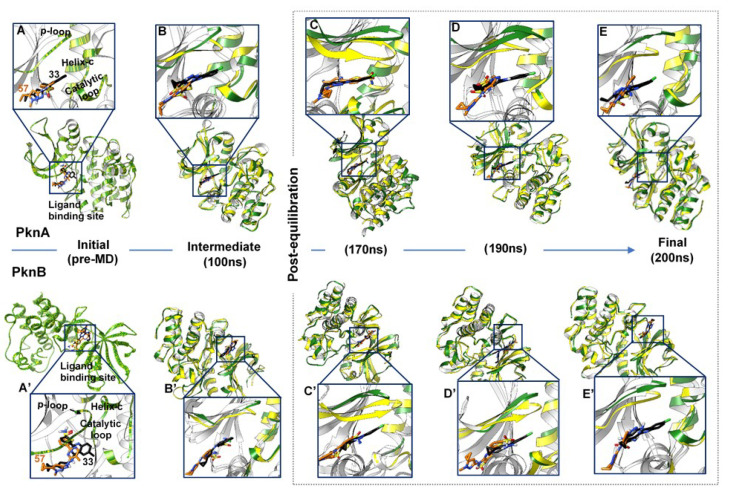
Comparative analyses of systemic alterations induced by the binding activities of compounds **33** (black) and **57** (orange) in PknA- and PknB-NBPs at different time-frames of the simulation run starting from their pre-MD complexes where no alterations in catalytic components occurred yet. Relative motions of PknA P-loop, catalytic loop and helix C regions are revealed in (**A**–**E**) from the pre-simulation to the post-equilibrated timeframes while relative alterations in PknB catalytic domain are showed in (**A’**–**E’**). Orientations of the compounds were also shown along the trajectories.

As shown in Figure 7, a steady downward pull of the P-loop region characterized the binding of compound **33** across the simulation periods. This was also accompanied by movements in the DFG motif, catalytic loop and helix C regions. These structural variations were also observed across the post-equilibrated time frames (170–200 ns). Consequentially, these motions (inward pulling effects) could complement the compactness of the NBP which in turn favored ligand stability. Comparatively, time-based structural analyses of the unbound PknA and B proteins revealed minimal variations among these components over the simulation period, particularly at the post-equilibrated trajectories (Supplementary Figure S4). 

**Figure 8:**
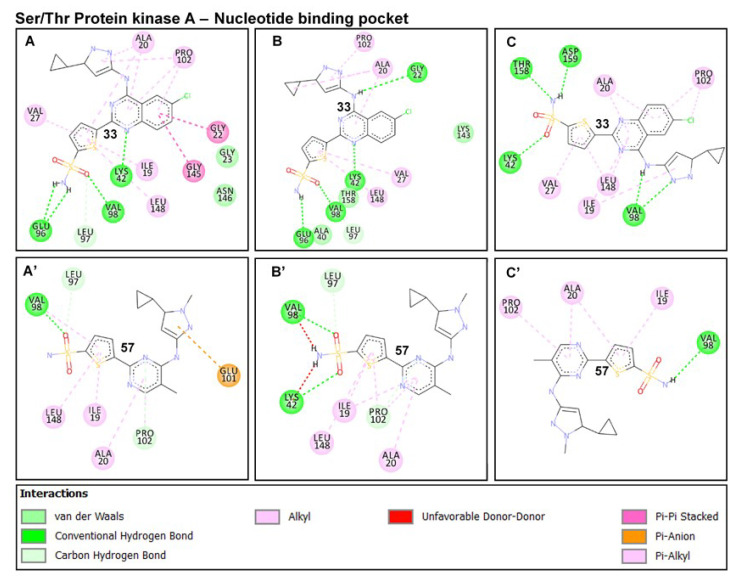
Comparative binding dynamics of compounds **33** and **57** at the NBP of PknA across post-equilibrated trajectories 170-200ns (**A**) 33-PknA at 170 ns. (**B**) 33-PknA at 190 ns (**C**) 33-PknA at 200ns. (**A’**) 57-PknA at 170 ns. (**B’**) 57-PknA at 190 ns. (**C’**) 57-PknA at 200 ns. Favorable and unfavorable interactions that characterize the binding of the ligands are shown and are well represented by their respective legends.

An in-depth look at the interaction mechanisms of **33** revealed key residues that contributed to high-affinity and stable binding, as analyzed from the terminal (post-equilibrated) time frames. These include GLY22 (→ GLY20_PknB_), LYS42 (→ LYS40_PknB_), GLU96 (→ GLU93_PknB_), VAL98 (→ VAL95_PknB_), LYS143 (→ LYS140_PknB_), THR158 (→ MET155_PknB_) and ASP159 (→ ASP156_PknB_) (Figure 8). GLY22 is located at the P-loop region, and its amide-π (n → π*_Ar_) interaction could contribute notably to the stability of compound **33**. The importance of this interaction with respect to the stability of small molecules has been previously reported [51]. Another important interaction observed was a high-affinity H-bond between the quinazoline ring of **33** and LYS42; one of the conserved catalytic residues of Ser/Thr kinases. This also could have played a crucial role in the stability of the compound. The terminal sulfonamide oxygen atom (O_1_) of compound **33**, as well, elicited conventional (NH---O) bond with VAL98 while an NH---O bond occurred between the carbonyl oxygen of GLU96 and terminal hydrogen (H_14_) of the compound. Moreover, these H-bonds have distances between 1.9–2.5 Å, indicative of their strengths and stabilities.

Conventional H-bond interactions elicited by THR158 and ASP159 with the sulfonamide group of **33** further contributed to its high-affinity binding stability at the active pocket. The occurrence of these interactions at the final time frames of simulation could suggest that the sulfonamide moiety was optimally displaced away from GLU96 and VAL98 which are located at the entrance of the NBP. This could indicate that compound **33** was more favorably oriented deep into the pocket for a more stable binding (see Figure 3A’,B’ above).

Also observed is the intermittent carbon hydrogen bond (CH---Cl) between the constituent Cl atom of compound **33** and LYS143 (catalytic loop), which could also contribute in part to the stability of the compound. This interaction was however lost in **57**-PknA due to the Cl → CH_3_ substitution. Additionally, residues such as ALA20, VAL27, PRO102 AND LEU148 interacted via π-alkyl interactions while LEU97 coordinately interacted with the sulfonic oxygen atom (O_1_) of **33**. 

These interactions occurred at the pyrazole and thiophene rings of the compound. In all, three residues of the P-loop; ALA20, GLY22, and VAL27 engaged in complementary interactions with compound **33** which could possibly explain the downward pull motions exhibited by the P-loop over the simulation period (see Figure 7).

**Figure 9:**
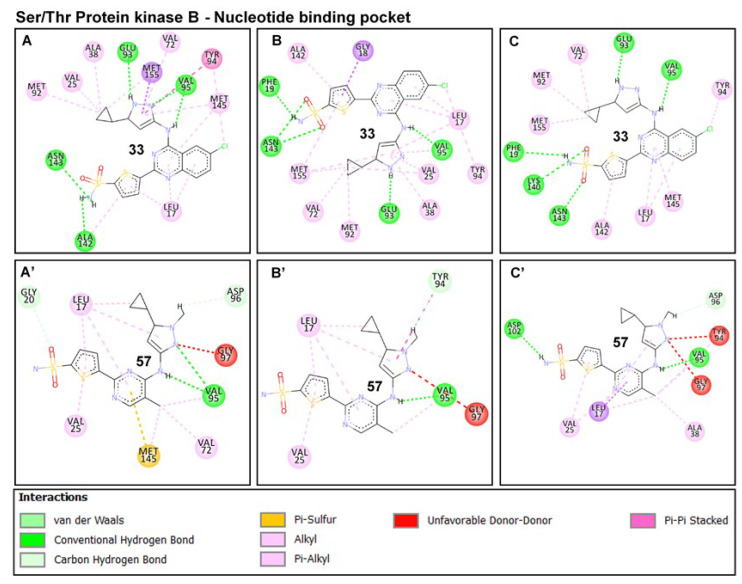
Binding dynamics of compounds **33** and **57** at the NBP of PknB as estimated from the post-equilibrated time frames 170–200 ns. (**A**) **33**-PknB at 170 ns. (**B**) **33**-PknB at 190 ns. (**C**) **33**-PknB at 200 ns. (**A’**) **57**-PknB at 170 ns. (**B’**) **57**-PknB at 190 ns. (**C’**) **57**-PknB at 200 ns. Favorable and non-favorable interactions involved disparately in the binding of **33** and **57** are indicated. Appropriate legends representing each interaction types are also shown.

For PknB, conventional hydrogen bonds characterized the binding of compound **33** in addition to a high number of aromatic (π) interactions. **33** interacted with PHE19, GLU93, and VAL95 via high-affinity NH---O bonds while LYS140 and ASN143 engaged with the terminal sulfonic O atoms of **33** (Figure 9). The π interactions could also play important roles in the stability of the compound since they occurred with the constituent thiophene, pyrazole, and quinazoline rings. On the contrary, **57** exhibited different interaction mechanisms that could possibly explain its inhibitory inactivities towards PknA and PknB. At the initial time frame (post-equilibrated − 170 ns), we observed that weak pi-alkyl interactions were more prominent in addition to minimal conventional H-bonds (NH--O) with VAL98 AND GLU96. 

An important highlight of the **57-**PknA complex was the occurrence of unfavorable donor-donor (NH---HN) interactions with VAL98 and LYS42, indicative of an unfavorable and unstable binding pattern. These unfavorable interactions could affect its binding at the deep hydrophobic region of the NBP of both proteins which could, in turn, explain its surface-binding as observed in Figure 3. Moreover, these interactions were intermittent and reduced at the final frame where scantly weak interactions were observed. Although **57** engaged in NH---O interactions with the carbonyl oxygen of PknB-VAL95 (VAL98 in PknA), unfavorable interactions occurred with TYR94 and GLY97 via its constituent pyrazole ring. Taken together, we could deduce that the occurrence of unfavorable interactions could underlie repulsive electrostatic effects on the binding of **57** at the NBPs of PknA and PknB.

**Figure 10:**
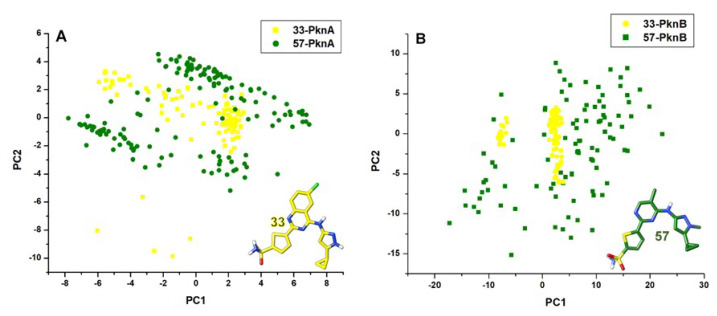
Principal component analyses of the differential motions of compounds **33** and **57** at the nucleotide binding pockets of (**A**) PknA and (**B**) PknB over the simulation period. Depicted in yellow and green colorations are compounds **33** and **57** respectively.

The differential stability and positioning of the compounds at the active pockets were further examined by the PCA and SASA values since the latter parameter is sufficient to predict the degree to which the compounds are exposed to the surrounding solvent [52]. We clustered the ligand motions along two principal components; PC1 and PC2 and observed that **57** demonstrated a more dispersed motion at the NBPs while a more compact motion was demonstrated by **33** (Figure 10). This further implies that compound **33** had a more stable binding compared to **57**. The unstable pattern of motion exhibited by **57** could suggest its inability to maintain high-affinity interactions with crucial residues at the NBP, hence the gradual loss in interaction as shown in Figure 8A’–C’ and Figure 9A’–C’.

Compounds **33** and **57** were then masked to further investigate their motions at the solvent phases and results revealed that **33** exhibited lower solvent motions indicative of its deep hydrophobic motions compared to **57** with high SASA peaks that could indicate its highly unstable and surface- motion, corroborative of findings earlier mentioned. Mean SASA values for **33** were 74.7 Å^2^ and 61.9 Å^2^ in PknA, while 146.2 Å^2^ and 101.2 Å^2^ were estimated for **57** in PknB. This corroborates findings presented in Figure 3A’ and 3B’ which showed that **33** was more deeply bound in the hydrophobic pockets of PknA and PknB while **57** was surface-bound, outside the NBP. Taken together, we could deduce from our findings that the highly unstable dynamics exhibited by **57** could account for its inability to bind dually and proximally at the NBPs of both PknA and B. This, in turn, could rationally explain its highly reduced affinity as experimentally reported.

### 2.4. Estimation of Binding Affinities and Per-Residue Energy Decomposition

The MM/GBSA-based approach was further used to estimate the differential binding of both compounds while the energy contributions of interacting residues were also determined. Findings are presented in Table 3. From our calculations, binding free energy (Δ*G_bind_*) increased from **33** to **57** by −25.72 kcal mol^−1^ in PknA and −29.04 kcal mol^−1^ in PknB. This further reflects the experimentally reported data in which **33** exhibited more favorable binding to both proteins compared to **57**. More so, entropical effects were minimal on the total binding energies and as shown, van der Waals (vdW) and electrostatic interactions in the gas phase were more favorable for **33** with a relative reduction for **57**. Also, we can deduce that, relative to electrostatic effects, vdW energies contributed more towards the binding and stability of **33** in both proteins. This could be, in part, due to the prominent π interactions earlier reported.

In addition, desolvation effects accounted for unfavorable polar (Δ*G*_ele,sol(GB)_) and total polar solvation energies (Δ*G*_sol_). These could imply that the binding of compound **33** was more favorable in the active pocket relative to **57** as indicated by its estimated (Δ*G*_ele,sol(GB)_) and (Δ*G*_sol_) values. These corroborate the non-polar (Δ*G*_np,sol_) energies derived from the burial of the ligand away from the solvent accessible region. Δ*G*_np,sol_ values were more favorable for **33** compared to **57**, which could imply that the former was more deeply bound in the hydrophobic pocket regions of both proteins.

Comparatively, electrostatic energies increased by −13.46 kcal mol^−1^ between **33**- and **57**-bound PknAs while vdW contributions varied by −18.13 kcal mol^−1^. Also, in PknB, electrostatic and van der Waals energies differ by −4.16 kcal mol^−1^ and −21.23 kcal mol^−1^ respectively. This further emphasizes the importance of van der Waals contributions to the high-affinity binding and stability of **33** in PknB. Noteworthy, contributions of Δ*G*_ele,sol(GB)_ were largely unfavorable to the total binding energies. This could suggest that electrostatic interactions associated with ligand motions at the active site may not be strong enough to compensate fully for the huge desolvation penalties that characterized ligand binding. Comparatively, we observed a huge decrease in net electrostatic interactions to −4.23 kcal mol^−1^ in PknA and 0.28 kcal mol^−1^ in PknB. Presumably, complementary vdW contributions were sufficient to influence the dual inhibitory activities of **33**.

**Figure 11:**
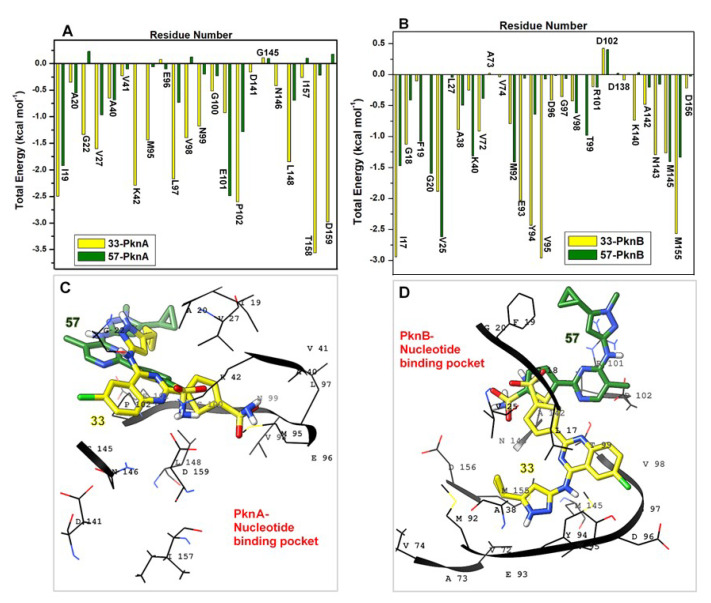
Total energy contributions of important catalytic residues to the differential binding and stabilities of compounds **33** and **57** at the nucleotide binding pockets of PknA and PknB. (**A**) Comparative energies of catalytic residues to **33** and **57** in PknA (**B**) Comparative energies of catalytic residues to **33** and **57** in PknB (**C**) Structural representation showing the positioning of **33** and **57** relative to the NBP residues in PknA (**D**) Structural representation showing the positioning of **33** and **57** relative to the NBP residues in PknB.

**Figure 12:**
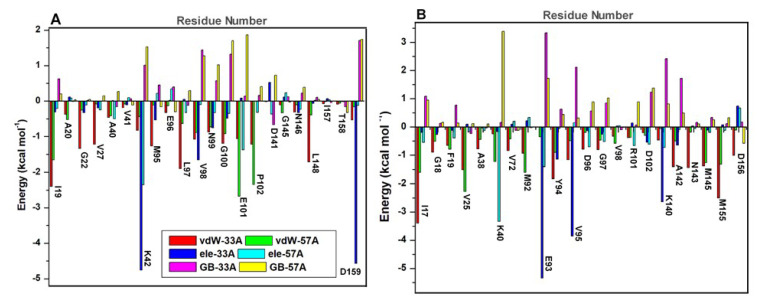
Energy (Δ*G_bind_*) decomposition on a per-residue basis into contributions from the van der Waals, electrostatic and polar solvation (ΔG_ele,sol (GB)_) energies for crucial NBP residues. (**A**) Δ*G_bind_* per-residue energy contributions of catalytic components in PknA nucleotide binding pocket (**B**) Per-residue energy contributions of catalytic components in PknB nucleotide binding pocket. Appropriate legends depicting the contribution type and protein system are shown likewise. vdW—van der Waals; ele—electrostatic; GB—polar solvation (ΔG_ele,sol (GB)_).

Binding free energies were further decomposed using the MM/GBSA method to identify crucial residues and their respective energy contributions to the disparate binding of both inhibitors. Per-residue interactions with energy contributions exceeding the −1 kcal mol^−1^ threshold were considered favorable [53]. Corresponding energy plots of interacting residues (within 5 Å radius) are shown in Figure 11 and Figure 12. In **33**-PknA; ILE19, GLY22, MET95, LEU97, VAL98, ASN99, PRO102, LEU148, THR158 and ASP159 showed energy contributions > −1 kcal mol^−1^, hence their presumed prominence towards the overall Δ*G_bind_* of compound **33**. These energies were decreased in the **57**- PknA with a majority of them falling below the −1 kcal mol^−1^ threshold. The crucial roles played by these residues via complementary hydrogen and aromatic interactions with the ligands (as earlier explained) are reflected by their energy contributions.

vdW energies from ILE19, GLY22, VAL27, MET95, LEU97 and LEU148 decreased from **33**-PknA to **57**-PknA by −0.74, −1.09, −1.14, −1.15, −1.27 and −1.23 kcal mol^−1^ respectively. Moreover, we could deduce that high involvement of aromatic interactions could contribute favorably to the high vdW energies estimated for the **33**-bound systems. Also, high vdW (−1.08 kcal mol^−1^) and electrostatic (−1.66 kcal mol^−1^) energies were contributed by VAL98 to the binding of **33**, which reduced by −0.2 kcal mol^−1^ (vdW) and −1.56 kcal mol^−1^ (electrostatic) in **57**-PknA. This could rationally explain the mechanistic loss of affinity exhibited by **57**. More so, PRO102 favored the binding of both **33** and **57** to PknA but energetically unfavorable contributions by ASP159 (Δ*E*_ele_ = 2.30 kcal mol^−1^) could exert negative effects on the overall Δ*G_bind_* of **57**. 

Comparatively, while vdW energetic contributions to **33** (> −1 kcal mol^−1^) added up to −13.05 kcal mol^−1^, the sum of electrostatic energies equaled −16.24 kcal mol^−1^, which was compensated to −10.71 kcal mol^−1^ by cumulative effects of unfavorable polar electrostatic effects (Δ*G*_ele,sol(GB)_). This further indicates that the binding and stability of **33** in PknA was also dependent on vdW forces with terms mainly associated with the occurrence of complementary π interactions. Noteworthy, unfavorable polar energies for **57** added up to 11.75 kcal mol^−1^, which drastically reduced occurring electrostatic effects (> −1 kcal mol^−1^) to 8.02 kcal mol^−1^. This provides additional clue into the unfavorable binding of **57** to PknA.

Further decomposition of energies between **33**- and **57**-bound PknBs, revealed a PknA-similar pattern of energetic contributions by the interacting residues. Residues such as LEU17 ( → ILE19_PknA_), GLY18 (→ ALA20_PknA_), PHE19 (→ THR21_PknA_), GLY20 (→ GLY22_PknA_), VAL25 (→ VAL27_PknA_), MET92 (→ MET95_PknA_), GLU93 (→ GLU96_PknA_), TYR94 (→ LEU97_PknA_), VAL95 (→ VAL98_PknA_), ASN143 (→ ASN146_PknA_), MET145 (→ LEU148_PknA_) and MET155 (→ THR158_PknA_) contributed highly favorable energies (> −1 kcal mol^−1^) to the binding and stability of **33**. Comparatively, respective energy decreases of −1.48, −0.72, −0.98, −0.75, −1.74, −0.62, −1.97, −1.79, −2.89, −1.14, −0.12 and −1.23 kcal mol^−1^ were estimated for these same residues in the **57**-PknB complex. Highest total energetic contributions to **33** in PknB were mediated by LEU17 (−2.95 kcal mol^−1^) and VAL95 (−2.96 kcal mol^−1^). Strong hydrogen bonds from VAL95 underlies its high vdW contributions while vdW energies from LEU17_PknB_ emanates from aromatic π alkyl interactions with which it coordinates the thiophene and quinazoline ring of **33** (Figure 9). Common to the binding of **33** and **57** is the unfavorable energy contributions by D102 which may arise from steric effects. As seen in Figure 11D, their sulfonamide Os seem to orient towards an O group in D102, although at different distances (closest in **33**). These orientations may elicit unfavorable O-O interactions, which would however be compensated for by other favorable energies relatively high in the **33**-bound complex. 

Correlative residues in PknA; ILE19 AND VAL98 (conserved), had similar high energy values of −2.40 and −1.08 kcal mol^−1^ respectively. Therefore, we could deduce that **33** interacted similarly with key active site residues, although stronger in PknB, which could account for higher affinity according to experimental data. Favorable vdW contributions to the binding and stability of **33** in PknB summed up to −17.60 kcal mol^−1^ which were cumulatively decreased to −7.28 kcal mol^−1^ in the presence of **57**. Highest vdW contributions to **33** were mediated by LEU17 (−3.40 kcal mol^−1^) and MET155 (−2.51 kcal mol^−1^) with reductions to −1.60 kcal mol^−1^ and −1.319 kcal mol^−1^ in the presence of **57**.

High electrostatic contributions of −5.35, −3.86, −2.64, −1.15 and −1.00 kcal mol^−1^ were contributed by GLU93 (→ GLU96_PknA_), VAL95 (→ VAL98_PknA_), LYS140 (→ LYS143_PknA_), TYR94 (→ LEU97_PknA_) and ASP138 (→ ASP141_PknA_) respectively, bringing the favorable energy (electrostatic) contributions to −14.00 kcal mol^−1^ (Figure 12). On the contrary, in **57**-PknB, electrostatic energy contributions by these residues were reduced to −1.40 kcal mol^−1^ (GLU93), 0.15 (VAL95), −0.72 (LYS140), −0.06 (TYR94) and −0.36 (ASP138). This notable difference could further support the superior binding affinity exerted by **33** to PknB relative to compound **57**. High electrostatic terms of GLU93 could have been partly due to its strong H-bond interactions while π interactions with the aromatic ring of TYR94 correlate with its high electrostatic contributions to **33**. Polar solvation terms were unfavorably (> 1 kcal mol^−1^) high in **57** with a total Δ*G*_ele,sol(GB)_ sum of 11.05 kcal mol^−1^ which was reduced to 7.17 kcal mol^−1^ in **33**. While compensatory effects render the total electrostatic contributions to **57** unfavorable (4.55 kcal mol^−1^), electrostatic contributions to **33** decreases to −6.83 kcal mol^−1^, further indicating the importance of the vdW contributions to the active site affinity and stability of **33** in PknB.

Taken together, the dual binding of **33** to PknA and PknB was cumulatively favored by van der Waals and electrostatic contributions from key core residues (conserved and non-conserved) at the hydrophobic NBPs of both proteins. On the other hand, unfavorable interactions drastically reduced the dual binding affinities of **57** at the protein binding pockets.

## 3. Computational Methods

### 3.1. Preparation of Ligand and Protein Starting Structures

The crystal structures of PknA and PknB were obtained from the RCSB Protein Data Bank (PDB) with entries 6B2Q and 6B2P respectively [26]. The retrieved structures were prepared by removing co-crystallized molecules not relevant to this study using UCSF Chimera software version 1.11.2. However, prior to the removal of co-crystallized compounds, three-dimensional (x, y, z) grid boxes were set to define corresponding coordinates correlative to active site regions (nucleotide binding pockets) in both proteins. This was necessary to ensure **33** and **57** were docked in the appropriate regions relative to the crystallized complexes. 2D structures of compounds **33** and **57** were prepared on MarvinSketch GUI while full energy optimization and minimization were done at the B3LYP/6-311++G(d,p) level using the Gaussian16 program package [54]. This was essential in order to obtain the most stable conformations for docking simulations. 

### 3.2. Docking and MD Simulations

Optimized structures of **33** and **57** were respectively docked into the binding regions of PknA and PknB using the UCSF Chimera-integrated Vina module. Prior to docking simulations, we ensured that the selected binding regions in both proteins entailed residues located within a 5.0 Å radius of the co-crystallized compounds. This was essential so as not to omit residues that might be crucially involved in ligand activity. Ten conformations were generated for each inhibitor and the best were preferentially selected based on their structural alignment with the co-crystallized compounds. This was to ensure that the co-crystallized ligand conformations were selected as initial structures for inhibitors **33** and **57** at the respective binding regions. Based on crystal orientations, **33**-PknA, **33**-PknB, **57**-PknA and **57**-PnkB complexes were selected as the docking-predicted models for MD simulations.

We performed long-timescale MD simulations using the Graphic Processor Unit (GPU) version of Amber18 together with its integrated modules [55]. Parameters were defined for the protein using the FF14SB forcefield while antechamber and parmchk modules were used to generate parameter files for the compounds. In order to define coordinate and topology files for the ligand-protein complexes, we executed the LEAP program, which also simultaneously performed system neutralization (by addition of counter Na+ and Cl− ions) and explicit solvation in a 10 Å sized TIP3P water box. Initial minimization was performed for 2500 steps using a 500 kcal mol^−1^. Å^2^ restraint potential after which a full minimization was carried out for 10000 steps with no restraints. The systems were then heated in a canonical (NVT) ensemble with a 5 kcal mol^−1^ Å^2^ harmonic restraint from 0–300 K for 50 ps with the aid of a Langevin thermostat [56]. This was followed by the equilibration step in an NPT ensemble for 1000 ps without restrains at a temperature of 300 K while the Berendsen barostat was used to maintain the pressure at 1bar [57]. The production run lasted for a period of 200 ns for each protein in their unbound and ligand-bound forms. These altogether consists of 6 systems: unbound PknA, unbound PknB, **33**-PknA, **33**-PknB, **57**-PknA and **57**-PknB. Resulting trajectories were captured at every 1ps after which they were analyzed as data plots on the Microcal Origin software version 6.0 [58]. Also, snapshots were retrieved for both complexes and were analyzed for conformational variations and interaction dynamics using the UCSF Chimera version 1.11.2 GUI and Discovery Studio 2016 Client [59].

### 3.3. Binding Energy Calculations and Decomposition of Per-Residue Energy

The non-uniformity in the binding affinities of both analogs to PknA and PknB were determined using the Molecular Mechanics/Generalized Born Surface Area (MM/GBSA) method. This was achieved by estimating their binding energies profiles which incorporates all energy components involved in complex formation. To minimize entropical effects, we retrieved 1000 snapshots from the final 30 ns MD trajectories where the systems stabilized. 

Binding energy calculations are represented mathematically as follows:

∆G_bind_ = G_complex_ − (G_receptor_ + G_inhibitor_) (1)

∆G_bind_ = ∆G_gas_ + ∆G_sol_ − T∆S = ∆H − T∆S (2)

∆G_gas_ = ∆E_int_ + ∆E_ele_ + ∆E_vdW_ (3)

∆G_sol_ = ∆G_ele,sol(GB)_ − ∆G_np,sol_ (4)

∆G_np,sol_ = γSASA + β (5)

From the above equations, the gas phase energy (∆G_gas_) is defined by the summation of internal (∆E_int_), electrostatic (∆E_ele_) and van der Waals (∆E_vdW_) energies while the polar solvation (∆G_ele,sol_) and non-polar contribution to solvation (∆G_np,sol_) terms define the solvation free energy, defined as ∆G_sol_. The Generalized Born (GB) model was solved with the MM/GBSA method to determine (∆G_ele,sol_) while as shown in eqn. (5), ∆G_np,sol_ was solved by a linear relationship between the surface tension proportionality constant (γ), solvent accessible surface area, SASA (Å^2^), and β which is also a constant. The value for γ is set at 0.0072 kcal (mol^−1^ Å^−2^) while the linear incorporation of pairwise overlaps (LCPO) model is used for SASA estimations.

In addition, we performed per-residue energy decomposition to obtain significant insights into the dynamic roles and energy contributions of site residues of PknA and PknB towards ligand-receptor binding and stabilization.

### 3.4. Analyses of Dynamic Cross-Correlation Matrix (DCCM)

This method was used to investigate dynamical movements and fluctuations of PknA and PknB C-α atoms in the presence of both compounds; **33** and **57**. This allows us to obtain structural insights into the time-correlated motions of residues within the protein systems over the simulation period, presented as a 3D matrix. DCCM is mathematically expressed as follows:

C_ij_ = < ∆r_i_ × ∆r_j_ > ÷ (< ∆r_i_^2^ > < ∆r_j_^2^ >)^1/2^ (6)

From the equation, C_ab_ depicts the mean positional displacement of the ath atom, with the angular brackets indicating the time average over the entire trajectory. a and b represent the ath and bth atoms while their respective displacements are shown as Δra and Δrb. The cross-correlation function varied from −1 to 1 depictive of the directions of motions. A highly positive C_ab_ value indicates motions that strongly correlated between residues a and b (same direction) while a negative value for C_ab_ shows that the direction of motion is anti-correlated. The CPPTRAJ module of Amber18 was used to obtain matrices from the trajectories which were plotted using the Origin analytical software version 6.0.

### 3.5. Principal Component Analyses (PCA) 

We employed the PCA method to investigate changes in the ligand dynamics and conformations over the course of the simulation [60,61] relative to their differential inhibitory activities. The PCA metrics constitute the eigenvalues and eigenvectors, which respectively describe the magnitude and direction of motions. The first two principal components (PC1 and PC2) were computed using the Amber18 CPPTRAJ module while the conformational behaviors of the simulated systems were projected along the direction of these two components (ev1/PC1 vs ev2/PC2).

## 4. Conclusions

The highly disparate dual binding activity of compound **33** compared **57** in Ser/Thr kinases; PknA *(**K_i_***: < 8 → > 4000 nM) and PknB (***K_i_***: < 1 → > 4000 nM), as experimentally reported makes it imperative to investigate the underlying mechanisms. This explains the rationale of this study which was achieved using computational methods that entailed molecular dynamics simulations and MM/GBSA-based analysis of binding free energy. Our findings revealed that compared to **57**, the binding of **33** stabilized the conformations of both proteins and reduced their structural activities. The impact of **33** was also prominent at the active pockets of the proteins where it reduced mobilities and enhanced compactness. Its effect at these regions could be due to complementary interactions with key residues, which could, in turn, enhance its optimal stability and positioning. 3D structural analyses of the binding pockets revealed that the primary conformations of the P-loop, catalytic loop, helix C and DFG motif were systematically altered by **33** in order to bind optimally. 

The NBPs of PknA and B, on the other hand, were highly unstable in the presence of **57** with high Cα motions among constituent residues. Analysis of ligand mobility revealed that **33** was more stable in PknA and B with characteristic deep hydrophobic binding while **57** was highly unstable and more surface bound. 

Furthermore, a similar pattern of bond formation was observed for **33** in both proteins, involving common (conserved and non-conserved) residues of their NBPs. Aromatic (π), coupled with hydrogen interactions played crucial roles in the binding of **33**, particularly GLY22 (→ GLY20_PknB_), LYS42 (→ LYS40_PknB_), GLU96 (→ GLU93_PknB_), VAL98 (→ VAL95_PknB_), LYS143 (→ LYS140_PknB_), THR158 (→ MET155_PknB_) and ASP159 (→ ASP156_PknB_). The quinazoline ring, in addition to the thiophene and pyrazole rings of **33** was crucial to the formation of a high number of π and H- interactions. Likewise, the Cl substituent of **33** engendered interactions with the catalytic loop residue, LYS143; an interaction that was lost in the **57**-bound complexes. These interactions culminated in high vdW and electrostatic energy contributions with Δ*G_bind_* of compound **33** summing up to −45.75 kcal mol^−1^ for PknA and −50.0 kcal mol^−1^ for PknB. Unfavorable interactions were observed in the **57**-PknA and B complexes, which could have repulsive effects on ligand positioning and stability. Consequently, binding free energy estimations revealed a notable decrease in **57**-Δ*G_bind_* to −20.9 kcal mol^−1^ (PknA) and −20.03 kcal mol^−1^ (PknB). More so, polar solvation terms were highly unfavorable in **57** compared to **33** while vdW energetic contributions were pertinent to the dual binding and stability of compound **33**. These findings, altogether, revealed the superior dual binding affinity of **33** over **57**, and underlying mechanisms. Rational explanations from this study are expected to significantly aid the structure-based design of highly selective molecules with dual inhibitory activities towards Ser/Thr kinases; PknA and PknB.

## Figures and Tables

**Table 1:** Conformational analysis of protein kinases A and B complexed with compounds **33** and **57**. Estimated *K_i_* value is also indicated.

Post Equilibration	PknA	33-PknA	57-PknA	PknB	33-PknB	57-PknB
**Whole Structure**						
Whole RMSD	2.42 ± 0.16	1.40 ± 0.2	1.14 ± 0.11	2.01 ± 0.30	1.39 ± 0.25	1.44 ± 0.19
FE-RMSD (Å)	2.38 ± 0.35	1.33 ± 0.23	1.82 ± 0.13	1.95 ± 0.29	1.24 ± 0.20	1.45 ± 0.18
FE-RMSF (Å)	1.41 ± 0.69	0.89 ± 0.40	1.00 ± 0.42	1.11 ± 0.34	0.79 ± 0.46	1.05 ± 0.55
*K_i_* (*nM*) [26]		<8	>4000		<1	>4000
**Nucleotide Binding Pockets**						
FE-RMSD (Å)	2.31 ± 0.31	1.51 ± 0.17	2.00 ± 0.23	2.42 ± 0.11	1.59 ± 0.26	2.34 ± 0.43
FE-RoG (Å)	13.10 ± 0.28	9.27 ± 0.13	10.26 ± 0.11	12.34 ± 0.03	8.54 ± 0.09	11.93 ± 0.14

**Table 2:** RMSF analysis of structural components of the PknA and PknB NBPs in the presence of compounds **33** and **57**.

Structural Fluctuation (Å)						
Structural Components	PknA	33-PknA	57-PknA	PknB	33-PknB	57-PknB
P-loop	2.36 ± 0.25	1.50 ± 0.36	1.46 ± 0.36	2.14 ± 0.14	0.92 ± 0.22	1.14 ± 0.37
Helix C	1.59 ± 0.51	1.11 ± 0.30	1.35 ± 0.50	1.52 ± 0.43	0.88 ± 0.32	1.48 ± 0.57
Catalytic loop	0.88 ± 0.10	0.70 ± 0.12	0.71 ± 0.13	0.69 ± 0.06	0.48 ± 0.04	0.66 ± 0.22
DFG-motif	1.41 ± 0.14	0.55 ± 0.02	0.72 ± 0.10	1.01 ± 0.04	0.48 ± 0.02	0.92 ± 0.05

**Table 3:** Binding free energy profiling of **33-** and **57-** PknA and PknB complexes.

Binding Free Energy Analysis				
Energy Components (kcal mol^−1^)	33-PknA	57-PknA	33-PknB	57-PknB
∆*E*vdW	−46.38 ± 0.18	−28.25 ± 0.10	−51.02 ± 0.12	−29.79 ± 0.11
∆*E*ele	−24.64 ± 0.22	−11.18 ± 0.08	−29.15 ± 0.19	−24.99 ± 0.42
∆*G*gas	−71.02 ± 0.26	−39.43 ± 0.10	−80.17 ± 0.20	−54.78 ± 0.83
∆*G*ele,sol(GB)	28.36 ± 0.13	29.89 ± 0.05	31.70 ± 0.13	35.26 ± 0.45
∆*G*np,sol	−5.85 ± 0.01	−3.0 ± 0.01	−6.26 ± 0.01	−5.57 ± 0.07
∆*G*sol	22.51 ± 0.13	26.89 ± 0.05	25.44 ± 0.13	29.99 ± 0.39
∆*H*	−48.51 ± 0.16	−12.54 ± 0.06	−54.73 ± 0.14	−24.79 ± 0.52
*T∆S*	−2.76 ± 0.08	−7.46 ± 0.03	−4.73 ± 0.06	−3.89 ± 0.26
∆*G*bind	−45.75 ± 0.24	−20.03 ± 0.15	−50.0 ± 0.14	−20.9 ± 0.15
*Ki (nM)*	<8	>4000	<1	>4000

## Supplementary Materials

The following are available online, Figure S1: RMSD analyses of structural and NBP stability of (**A**) unbound PknA (black) and unbound PknB (red) (**B**) Comparative whole structure FE-RMSD (post-equilibrated) of unbound PknA and PknB (**C**) Comparative FE-RMSD analysis of unbound PknA and PknB nucleotide binding pocket (**D**) FE-RoG analysis of the degree of mobility (compactness) of unbound PknA and PknB. Figure S2: Hydrogen bond analyses of the simulated systems across the entire trajectory (**A**) unbound- (black), **57**- (green) and **33**-bound (red) PknA (**B**) unbound-, **57**- and **33**-bound PknB. Inset is the estimation of average hydrogen bonds that occurred within each system over the entire trajectory. Figure S3: Post-equilibrated (FE) RMSF analyses of unbound PknA and PknB. Highlighted in (**A**,**B**) are regions that correspond to the P-loop, Helix C, Catalytic loop and DFG motif. (**C**) DCCM analysis of residual motions across the entire unbound PknA structure and (**D**) DCCM analysis for unbound PknB. Highlighted are motions within the catalytic components. Figure S4: Conformational variations of catalytic components in unbound PknA and PknB along the simulated trajectories. Table S1. Hydrogen bond occupancies ≥90% in the unbound PknA, unbound PknB, 33-PknA, 33-PknB, 57-PknA and 57-PknB.
