# Supplementary material for: Probing the Highly Disparate Dual Inhibitory Mechanisms of Novel Quinazoline Derivatives against Mycobacterium tuberculosis Protein Kinases A and B
Source: Molecules. 2020 Sep 16;25(18):4247. doi: 10.3390/molecules25184247 (PMC7571077; doi:10.3390/molecules25184247)
Supplement: Supplementary file 1 [file molecules-25-04247-s001.pdf]

molecules

MDPI

Article

Probing the Highly Disparate Dual Inhibitory Mechanisms of Novel Quinazoline Derivatives Against *Mycobacterium tuberculosis* Protein Kinases A and B

Fisayo A. Olotu and Mahmoud E. Soliman *

Molecular Bio-computation and Drug Design Laboratory, School of Health Sciences, University of KwaZulu-Natal, Westville Campus, Durban 4001, South Africa; olotuf@ukzn.ac.za

* Correspondence: soliman@ukzn.ac.za; Tel.: +27 (0) 31 260 8048; Fax: +27 (0) 31 260 7872

SUPPLEMENTARY INFORMATION

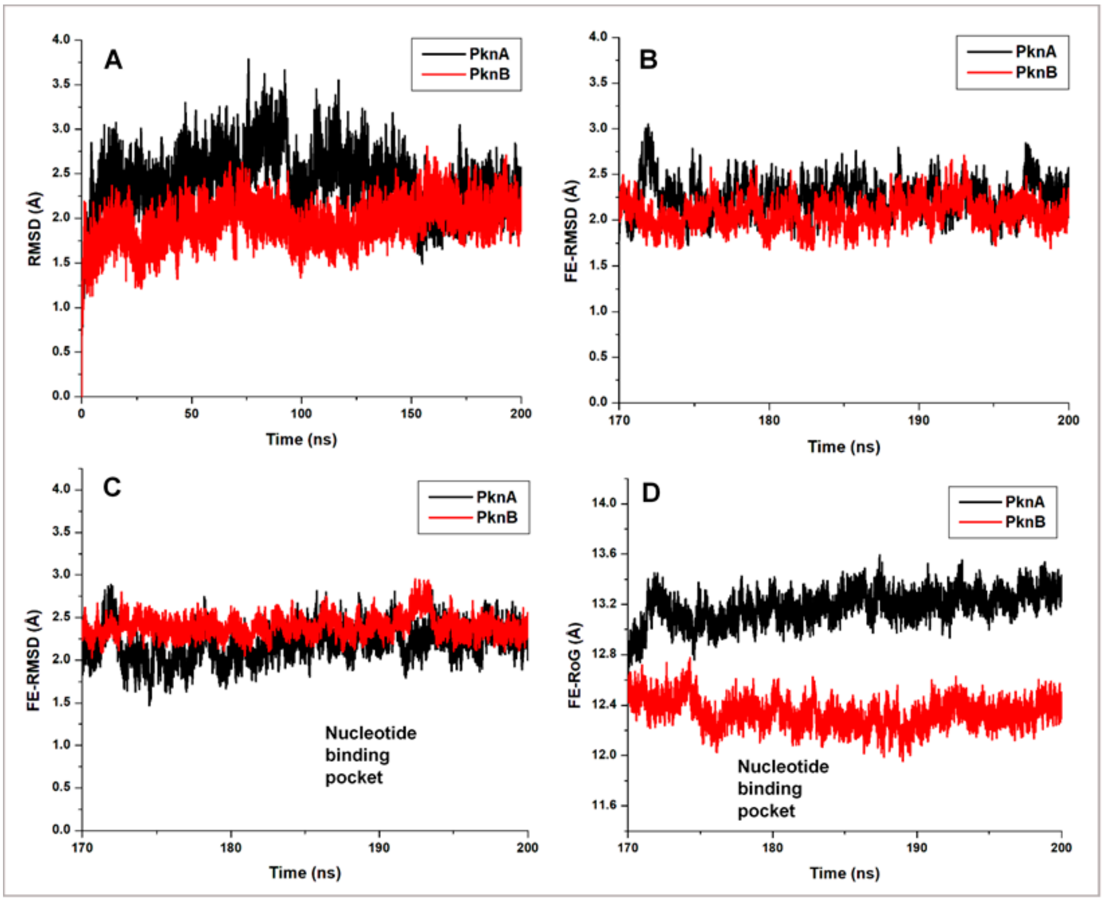

Figure S1: RMSD analyses of structural and NBP stability of (**A**) unbound PknA (black) and unbound PknB (red) (**B**) Comparative whole structure FE-RMSD (post-equilibrated) of unbound PknA and PknB (**C**) Comparative FE-RMSD analysis of unbound PknA and PknB nucleotide binding pocket (**D**) FE-RoG analysis of the degree of mobility (compactness) of unbound PknA and PknB.

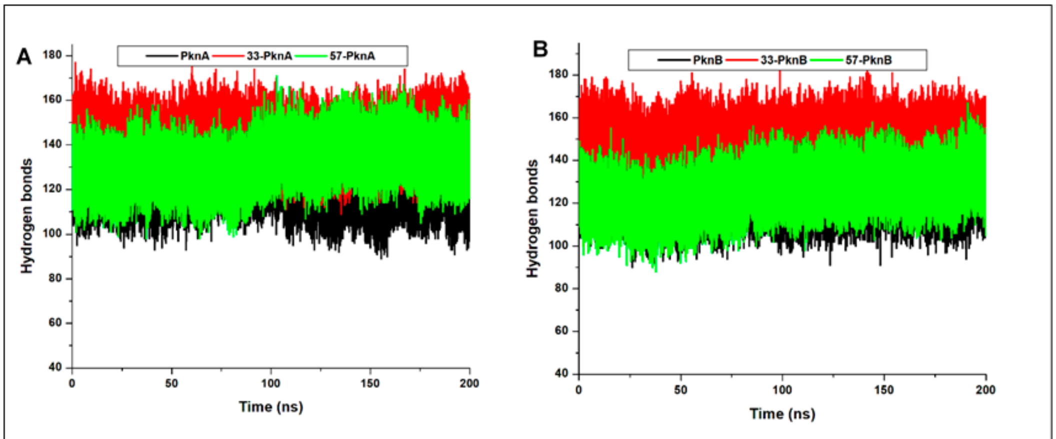

	PknA	PknB	33-PknA	33-PknB	57-PknA	57-PknB
Hydrogen bonds	122±0.02	123±0.16	143±0.11	149±0.27	132±0.19	126±0.28

Figure S2: Hydrogen bond analyses of the simulated systems across the entire trajectory (**A**) unbound- (black), **57**- (green) and **33**-bound (red) PknA (**B**) unbound-, **57**- and **33**-bound PknB. Inset is the estimation of average hydrogen bonds that occurred within each system over the entire trajectory.

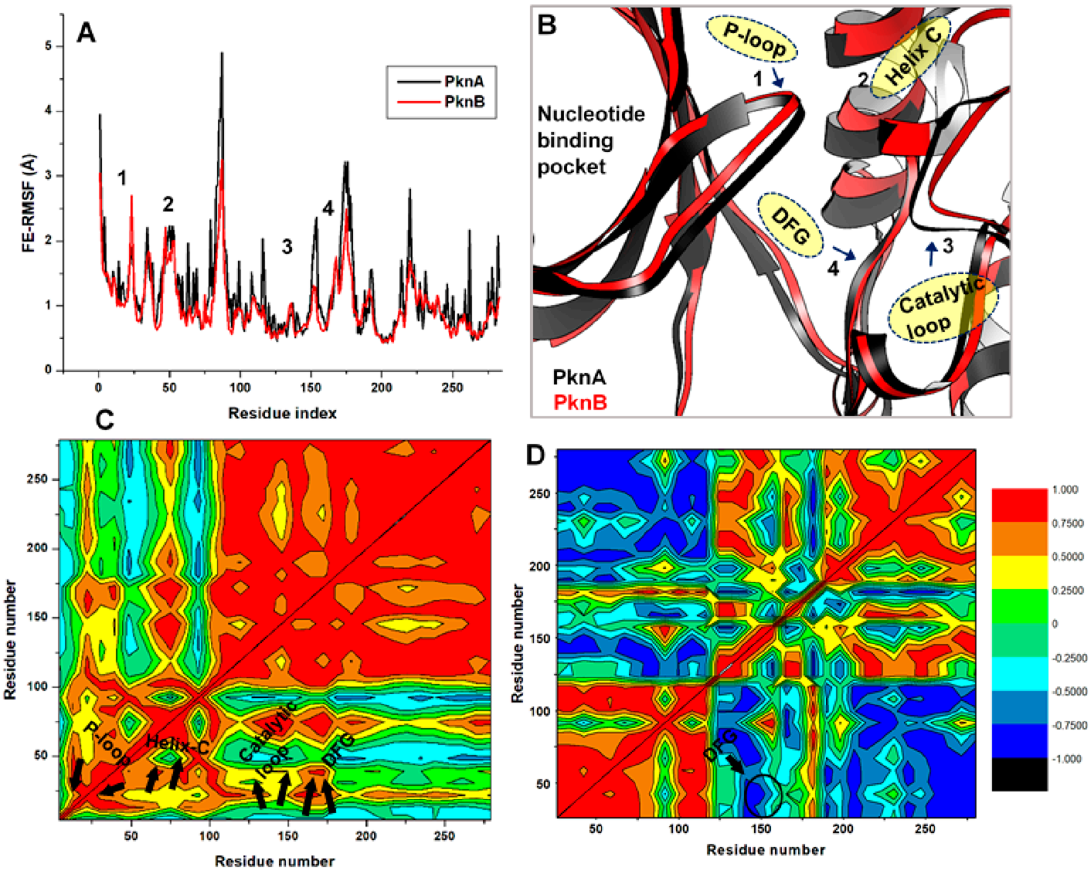

Figure S3: Post-equilibrated (FE) RMSF analyses of unbound PknA and PknB**.** Highlighted in (**A**) and (**B**) are regions that correspond to the P-loop, Helix C, Catalytic loop and DFG motif. (**C**) DCCM analysis of residual motions across the entire unbound PknA structure and (**D**) DCCM analysis for unbound PknB. Highlighted are motions within the catalytic components.

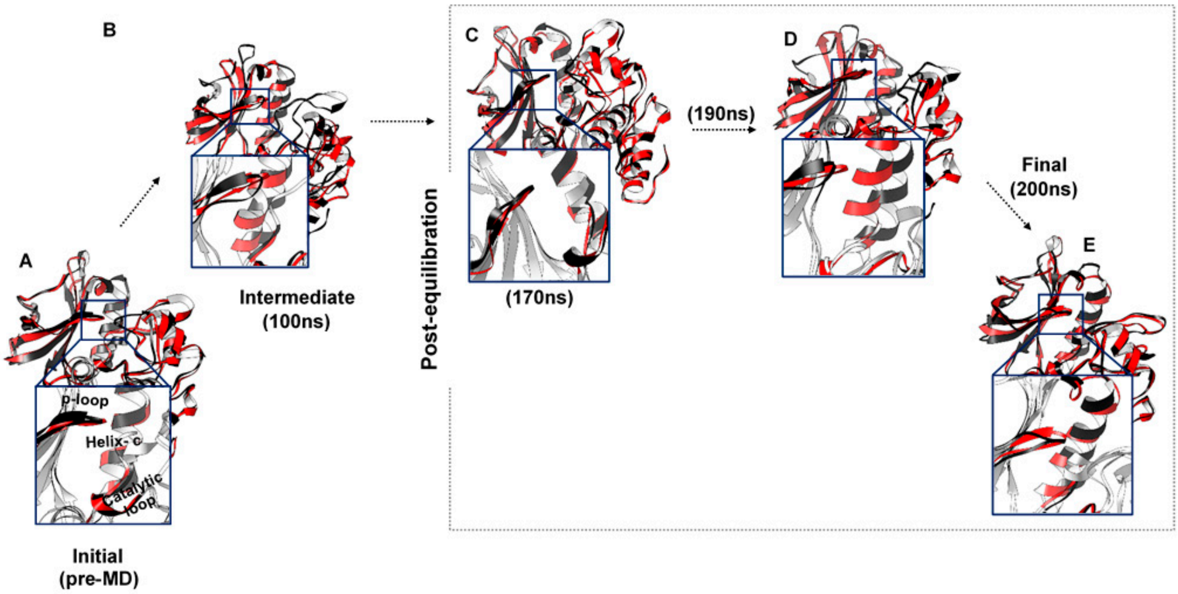

Figure S4: Conformational variations of catalytic components in unbound PknA and PknB along the simulated trajectories.

Table 1. Hydrogen bond occupancies ≥90% in the unbound PknA, unbound PknB, 33-PknA, 33-PknB, 57-PknA and 57-PknB.

Systems	H-Acceptor	H-Donor	Donor	Occupancy (%)	Distance (Å)	Angle (°)
PknA	GLU187-OE2	ARG260-HH12	ARG260-NH1	97.10	2.8	165
	LEU248-O	THR252-HG1	THR252-OG1	96.18	2.7	163
	MET121-O	THR125-HG1	THR125-OG1	95.20	2.7	163
	ASP199-OD2	HIS139-H	HIS139-N	93.40	2.8	161
	PRO281-O	TYR261-HH	TYR261-OH	93.34	2.7	165
	GLU187-OE1	ARG260-HH22	ARG260-NH2	93.20	2.8	164
	TYR183-O	SER202-HG	SER202-OG	92.4	2.7	165
PknB	GLU166OE2	ARG242-HH22	ARG242-NH2	97.12	2.8	165
	ILE214-O	TYR187-HH	TYR187-OH	95.71	2.7	166
	TYR162-O	SER181-HG	SER181-OG	95.54	2.7	164
	ASP30-OD2	ARG10-HH11	ARG10-NH1	93.92	2.8	160
	GLU166-OE1	ARG242-HH12	ARG242-NH1	93.90	2.8	166
33-PknA	GLU187-OE2	ARG260-HH12	ARG260-NH1	98.25	2.8	164
	TYR183-O	SER202-HG	SER202-OG	95.53	2.7	164
	GLU187-OE1	ARG260-HH22	ARG260-NH2	95.12	2.8	164
	MET121-O	THR125-HG1	THR125-OG1	94.75	2.7	164
	ARG17-O	GLU29-H	GLU29-N	92.69	2.8	164
	LEU248-O	THR252-HG1	THR254-OG1	92.38	2.7	163
	ASP199-OD2	HIS139-H	HIS139-N	91.96	2.8	164
	ASP32-OD1	ARG12-HE	ARG12-NE	91.91	2.8	164
	PRO281-O	TYR261-HH	TYR261-OH	91.50	2.7	165
	GLU29-O	GLN16-H	GLN16-N	91.27	2.8	160
	GLU61-O	THR65-HG1	THR65-OG1	90.22	2.7	163
	ASP32-OD2	ARG12-HH21	ARG12-NH2	90.11	2.8	159
	GLU187-OE1	GLU187-H	GLU187-N	90.08	2.8	151
	ASP199-OD2	HIS133-HE2	HIS133-NE2	90.03	2.8	166
33-PknB	ASP228-OD2	SER217-HG	SER-217-OG	98.20	2.6	166
	GLU188-OE1	TYR162-HH	TYR162-OH	96.72	2.6	166
	GLU166-OE1	ARG242-HH12	ARG242-NH1	95.40	2.8	165
	ILE134-O	SER129-HG	SER129-OG	95.28	2.7	164
	ILE214-O	TYR187-HH	TYR187-OH	95.21	2.7	166
	ASP30-OD2	ARG10-HH11	ARG10-NH1	92.66	2.8	159
	ASP76-O	VAL91-H	VAL91-N	92.24	2.8	161
	ALA38-O	MET92-H	MET92-N	92.17	2.8	162
	GLU166-OE2	ARG242-HH22	ARG242-NH2	92.11	2.8	164
	VAL91-O	TYR75-H	TYR75-N	92.08	2.8	160
	MET145-O	LYS153-H	LYS153-N	92.06	2.8	161
	VAL25-O	LEU17-H	LEU17-N	92.01	2.8	157
	HIS26-O	VAL39-H	VAL39-N	91.19	2.8	160
	ASP178-OD2	HIS136-H	HIS136-N	91.00	2.8	156
	GLU166-OE1	GLU166-H	GLU166-N	90.06	2.8	150
57-PknA	GLU187-OE2	SER198-HG	SER198-OG	98.23	2.6	167
	TYR183-O	SER202-HG	SER202-OG	97.07	2.7	164
	GLU187-OE2	ARG260-HH12	ARG260-NH1	96.70	2.8	164
	GLU187-OE2	ARG260-HH22	ARG260-NH2	95.53	2.8	165
	LEU248-O	THR252-HG1	THR252-OG1	95.02	2.7	164
	MET121-O	THR125-HG1	THR125-OG1	92.51	2.7	162
	PRO281-O	TYR261-HH	TYR261-OH	91.89	2.7	166
	ASP32-OD2	ARG12-HH21	ARG12-NH2	91.82	2.8	157
	ASP199-OD2	HIS139-H	HIS139-N	91.42	2.8	165
	PRO235-O	TYR208-HH	TYR208-OH	91.37	2.7	163
57-PknB	ASP228-OD2	SER217-HG	SER217-OG	98.67	2.6	167
	GLU166-OE2	ARG242-HH22	ARG242-NH2	96.36	2.8	165
	ILE214-O	TYR187-HH	TYR187-OH	95.05	2.7	166
	ASP30-OD2	ARG10-HH11	ARG10-NH1	93.43	2.8	160
	GLU166-OE1	ARG242-HH12	ARG242-NH1	92.97	2.8	166
	VAL25-O	LEU17-H	LEU17-N	92.77	2.8	155
	GLU166-OE1	GLU166-H	GLU166-N	92.61	2.8	149
	VAL91-O	TYR75-H	TYR75-N	91.74	2.8	163
	ALA38-O	MET92-H	MET92-N	91.62	2.8	159
	VAL39-O	HIS26-H	HIS26-N	90.16	2.9	160

© 2020 by the authors. Submitted for possible open access publication under the terms and conditions of the Creative Commons Attribution (CC BY) license (http://creativecommons.org/licenses/by/4.0/).
